# The Role of the Effects of Endoplasmic Reticulum Stress on NLRP3 Inflammasome in Diabetes

**DOI:** 10.3389/fcell.2021.663528

**Published:** 2021-04-14

**Authors:** Shuangyu Lv, Xiaotian Li, Honggang Wang

**Affiliations:** Bioinformatics Center, School of Basic Medical Sciences, Institute of Biomedical Informatics, Henan University, Kaifeng, China

**Keywords:** endoplasmic reticulum stress, NLRP3 inflammasome, diabetes, diabetic encephalopathy, diabetic nephropathy

## Abstract

Endoplasmic reticulum (ER) is an important organelle for the protein synthesis, modification, folding, assembly, and the transport of new peptide chains. When the folding ability of ER proteins is impaired, the accumulation of unfolded or misfolded proteins in ER leads to endoplasmic reticulum stress (ERS). The nucleotide-binding oligomerization domain-like receptor family, pyrin domain-containing 3 (NLRP3) inflammasome, can induce the maturation and secretion of interleukin-1beta (IL-1β) and IL-18 through activating caspase-1. It is associated with many diseases. Studies have shown that ERS can regulate NLRP3 inflammasome in many diseases including diabetes. However, the mechanism of the effects of ERS on NLRP3 inflammasome in diabetes has not been fully understood. This review summarizes the recent researches about the effects of ERS on NLRP3 inflammasome and the related mechanism in diabetes to provide ideas for the relevant basic research in the future.

## Introduction

Diabetes is characterized by the destruction of glucose homeostasis and the deficiency of insulin effect on the liver, muscle, pancreas, and fat (Sarwar et al., 2010; Defronzo et al., 2015). Diabetes can be divided into type 1 and type 2 diabetes. Type 1 diabetes accounts for about 5–10% of all diabetics, which is due to β-cell dysfunction, decreased insulin release, and decreased circulating insulin level. Type 2 diabetes is the most common type of diabetes, accounting for about 90–95% of all diabetics. It is mainly related to the insufficiencies of insulin response and insulin resistance of surrounding tissues (Amer Diabet, 2014). There were 366 million diabetics in 2011, and it is estimated that the total number of diabetics will increase to 552 million by 2030 (Whiting et al., 2011). Long-term diabetes can lead to multiple organ dysfunction, including kidney disease, retinopathy, neuropathy, atherosclerosis, and heart disease (Zheng et al., 2018; Inzucchi et al., 2019). Therefore, it is particularly urgent to find ways to treat and prevent diabetes complications.

Inflammasome, composed of many proteins, is an important part of the innate immune system and used to detect whether there are infections, pathogens, and metabolic alarms in cells (Elliott and Sutterwala, 2015; Jo et al., 2016). Inflammasomes have been identified to include NLRP1, NLRC4, RIG-I, AIM2, and NLRP3 (Barbe et al., 2014). NLRP3 inflammasome, the most deeply studied one, is composed of NLRP3, the adaptor protein ASC, and pre-caspase-1. It is mainly expressed in bone marrow cells, such as macrophages (O’connor et al., 2003). When the host is stimulated by the exogenous or endogenous stimuli, NLRP3 inflammasome is activated, leading to the recruitment of pre-caspase-1 and the adaptor protein ASC in macrophages. Stimulated NLRP3 interacts with pre-caspase-1 and the adaptor protein ASC to form a large cytoplasmic complex, thus activating caspase-1. Activated caspase-1 induces inflammation by cleaving pre-interleukin (IL)-1β and pre-IL-18 into IL-1β and IL-18 (Prochnicki et al., 2016). NLRP3 inflammasome is activated in two steps: the first signal (signal 1) indicating infection or tissue damage includes toll-like receptor 4, a pattern recognition receptor that can recognize lipopolysaccharide (LPS) and a series of endogenous risk signals, which activate nuclear factor kappa B (NF-κB) to increase the protein expression of NLRP3, pro-IL-1β, and pro-IL-18. Then, the second signal (signal 2), indicating cell damage, includes extracellular adenosine triphosphate (ATP), urate, and cholesterol crystals. It induces the assembly of inflammasome, activates caspase-1, and cleaves pre-IL-18 and pre-IL-1β into their active forms (Sokolova et al., 2019). The abnormal activation of NLRP3 inflammasome can lead to a variety of diseases including sterile inflammatory diseases (Abderrazak et al., 2015), diabetes (Luo et al., 2014), and non-alcoholic fatty liver diseases (Lv et al., 2019). In the hyperglycemic environment, the increase in NLRP3 inflammasome-dependent IL-1β secretion leads to the dysfunction of insulin secretion of β cells, promotes obesity and insulin resistance, and eventually leads to diabetes (Iannantuoni et al., 2019).

Endoplasmic reticulum (ER) is an important organelle, responsible for the protein synthesis, modification, folding, assembly, and transport (Han and Kaufman, 2016). In various stress states (including glucose deficiency, environmental toxins, viral infection, Ca^2+^ level changes, hypoxia, inflammation, and oxidative stress), ER homeostasis is destroyed, which triggers ER stress (ERS). ERS refers to the dysfunction of ER, which interferes with the protein folding, posttranslational modification, and secretion. The unfolded protein reaction (UPR) is initiated by the accumulation of unfolded protein in ER (Liu et al., 2017; Lan et al., 2019), which improves protein folding, promotes quality control mechanisms and degradation pathways, or activates apoptosis when the damage is irreversible (Hetz and Saxena, 2017). In addition to UPR, the ubiquitin–proteasome system (UPS) is another intracellular machinery for the unfolded protein degradation, in which the target proteins are first polyubiquitinated before being degraded (Caldeira et al., 2014). Under ERS, there are three parallel signaling pathways in UPR: pancreatic endoplasmic reticulum kinase (PERK)-mediated pathway, inositol-dependent enzyme 1 (IRE1)-mediated pathway, and activating transcription factor 6 (ATF6)-mediated pathway. Moderately activated ERS and UPR can promote the steady-state recovery of ER and help cells adapt to environmental changes. Under excessive and sustained stress, ERS and UPR can activate caspase-12 and CCAAT/enhancer-binding protein homologous protein (CHOP)-dependent apoptosis pathways and promote cell death, thus participating in the occurrence of various diseases (Ji et al., 2019). Studies have shown that ERS is closely related to NLRP3 inflammasome in many diseases including diabetes (Ji et al., 2019). However, the mechanism of the effects of ERS on NLRP3 inflammasome in diabetes has not been fully understood. In this review, the effects of ERS on NLRP3 inflammasome and its mechanism are explored in diabetes to provide ideas for the relevant basic research in the future.

## The Role of the Effects of ERS on NLRP3 Inflammasome in Endothelial Dysfunction of Diabetes

Vascular endothelial dysfunction is the main pathophysiological cause of diabetic vascular complications. Glucose toxicity plays a key role in the occurrence of diabetic endothelial dysfunction (Giaccari et al., 2009). Mangiferin, a xanthic acid, is found in mangoes and *Anemarrhena asphodeloides* Bunge. It has been widely used in traditional Chinese medicine for the treatment of diabetes by reducing blood sugar and improving blood lipid in patients with diabetes (Muruganandan et al., 2005; Marquez et al., 2012; Gong et al., 2013; Wang et al., 2014). Song et al. (2015) established ERS-related endothelial dysfunction model by using high glucose (HG) to stimulate endothelial cells and conducted a series of experiments. The results showed that in endothelial cells, the level of AMP-activated protein kinase (AMPK) phosphorylation was increased by Mangiferin. Mangiferin and AMPK activator (AICAR) inhibited IRE1α-mediated ERS and ROS production induced by HG, while Compound C, an AMPK inhibitor, abolished Mangiferin effects, suggesting that Mangiferin suppressed HG-induced ERS and oxidative stress by activating AMPK. Moreover, Mangiferin suppressed apoptosis, NLRP3 inflammasome activation, and the resultant increased level of IL-1β induced by HG, while compound C reversed the changes, indicating that Mangiferin suppressed HG-induced NLRP3 inflammasome activation and apoptosis of endothelial cells by activating AMPK (Song et al., 2015). Thioredoxin-interacting protein (TXNIP) connects ERS with NLRP3 inflammasome activation (Oslowski et al., 2012). Mangiferin and tauroursodeoxycholic acid (TUDCA, an ER stress inhibitor) suppressed HG-induced TXNIP expression, while Compound C reduced the inhibitory effects of Mangiferin on TXNIP expression. Collectively, it can be inferred that Mangiferin inhibited HG-induced TXNIP/NLRP3 inflammasome activation through suppressing ERS by activating AMPK to improve endothelial dysfunction (Song et al., 2015). In the above studies, the relationship among ERS, NLRP3 inflammasome, TXNIP, reactive oxygen species (ROS), and apoptosis needs to be further clarified.

## The Role of the Effects of ERS on NLRP3 Inflammasome in Adipose Dysfunction of Diabetes

In diabetes, hyperglycemia usually leads to chronic inflammation by increasing macrophage infiltration and oxidative stress in adipose tissue (Zeyda and Stulnig, 2007; Kawasaki et al., 2012). ERS can induce inflammation mediated by NLRP3 inflammasome (Lerner et al., 2012) and link inflammation and oxidative stress in the adipose tissue (Hummasti and Hotamisligil, 2010). Metformin, which can suppress inflammation, is an oral hypoglycemic agent for type 2 diabetes (Hattori et al., 2006). Resveratrol has strong antihyperglycemia, antioxidant, and anti-inflammatory effects (Chang et al., 2012). Both metformin and resveratrol improve adipose dysfunction by inhibiting inflammation and activating AMPK (Ashabi et al., 2015). In view of the above, in order to study whether metformin and resveratrol can improve adipose dysfunction by regulating ERS-induced NLRP3 inflammasome, Li and colleagues conducted a series of studies. The studies showed that metformin and resveratrol, as well as TUDCA, inhibited ROS generation and ERS induced by HG and improved glucose homeostasis in the adipose tissue of diabetic mice, indicating that ERS was involved in the inhibitory effects of metformin and resveratrol on HG-induced oxidative stress (Li et al., 2016). HG-induced oxidative stress could promote mitochondrial fission by activating dynamin-related protein1 (Drp1), a mitochondrial fission regulator (Smirnova et al., 2001), which was reversed by DPI (a cellular ROS inhibitor) and mitoTEMPO (a mitochondrial ROS inhibitor) in adipocytes or adipose tissue of diabetic mice, indicating that ROS mediated the mitochondrial fission induced by HG-induced oxidative stress. In addition, compound C and AMPK small-interfering RNA (siRNA) diminished the inhibitory effects of metformin and resveratrol on Drp1, suggesting that AMPK activation mediated the suppression of Drp1 by metformin and resveratrol. Metformin, resveratrol, and TUDCA also suppressed TXNIP/NLRP3 inflammasome by decreasing the induction of TXNIP, NLRP3, and cleaved caspase-1 and the IL-1β secretion in the adipose tissue of diabetic mice, suggesting that ERS mediated the inhibitory effects of metformin and resveratrol on HG-induced TXNIP/NLRP3 inflammasome. Moreover, adipocyte apoptosis was inhibited by metformin, resveratrol, TUDCA, and the anti-IL-1β antibody. Collectively, it can be inferred that metformin and resveratrol ameliorate adipose dysfunction through inhibiting ERS-induced NLRP3 inflammasome and Drp1-mediated mitochondrial fission, which may be an effective treatment strategy for adipose dysfunction in diabetes (Li et al., 2016). Enhancing AMPK could inhibit ERS (Song et al., 2015); therefore, whether AMPK mediates the effects of metformin and resveratrol remains to be studied. The interplay between Drp1-mediated mitochondrial fission and ERS also remains to be elucidated.

## The Role of the Effects of ERS on NLRP3 Inflammasome in Diabetic Encephalopathy

Diabetic encephalopathy including depression is one of the major complications of diabetes (Elamoshy et al., 2018; Han et al., 2018). Oxidative stress, ERS, and inflammation are involved in diabetic encephalopathy (Pei and Sun, 2018). Previous studies have shown that NLRP3 inflammasome is activated in the hippocampus of diabetic mice (Zhai et al., 2018) and is induced by ERS in obese rats fed with high fat diet (Cai et al., 2016). However, the link between NLRP3 inflammasome and ERS in diabetic encephalopathy is not clear. Ye et al. found that Gastrodin (Gas), one of the main bioactive components of rhizome of *Gastrodia elata*, could improve diabetes through reducing body weight, fasting blood glucose, dyslipidemia, and insulin resistance in diabetic mice. Gas also ameliorated depression-like behaviors by improving damaged neurons of diabetic mice. Mechanistic studies revealed that Gas decreased the expression levels of ERS-related proteins, NLRP3 inflammasome, and TXNIP and increased Bcl-2/Bax ratio in the hippocampus of diabetic mice, suggesting that Gas alleviated diabetic depression-like behaviors via inhibiting ERS-induced TXNIP/NLRP3 inflammasome, which need to be further studied (Ye et al., 2018a,b). Similar to the above, recent evidence suggests that hyperglycemia promotes oxidative stress and inflammation in the development of memory and cognitive impairment caused by diabetes (Miguel Roman-Pintos et al., 2016), while the inhibition of HG-induced NLRP3 inflammasome has protective effects on memory and cognitive impairment (Ye et al., 2018a; Zhai et al., 2018). Neferine (NE), suggesting that NLRP3 inflammasome is involved in diabetes-induced memory and cognitive impairment. Neferine (NE) is a unique dibenzylisoquinoline alkaloid from the seed embryo of lotus root and has a long history of application as a folk Chinese herbal medicine in China. NE has been reported to have anti-inflammatory effect (Jung et al., 2010; Asokan et al., 2018). The research of Wu et al. showed that NE ameliorated glucose tolerance, insulin sensitivity, dyslipidemia, memory impairment, and cognitive dysfunction in diabetic mice. Mechanistic studies revealed that NE inhibited oxidative stress and decreased the expression levels of TXNIP, NLRP3 inflammasomes, and ERS-related proteins in the hippocampus of diabetic mice (Wu et al., 2020). It can be inferred from the above that NE may ameliorate dyslipidemia, memory impairment, and cognitive dysfunction in diabetic mice by inhibiting ERS-induced TXNIP/NLRP3 inflammasome, which provides a treatment strategy for diabetic encephalopathy. The above inference needs to be further studied by using ERS activator and NLRP3 inflammasome inducer.

## The Role of the Effects of ERS on NLRP3 Inflammasome in Diabetic Retinal Injury

Retinal injury, which is characterized by chronic low-grade inflammation, is the most common complication of diabetes (Kern, 2007; Rossi et al., 2016). It has been reported that intact retina expresses hydroxycarboxylic acid receptor 2 (HCA2), which is activated by β-hydroxybutyrate (BHB). BHB, an endogenous ketone body, is produced by the oxidation of fatty acids in liver mitochondria when carbohydrate supply is insufficient (Martin et al., 2009; Gambhir et al., 2012; Dedkova and Blatter, 2014). The evidence demonstrate that HCA2 receptor has anti-inflammatory properties (Lukasova et al., 2011; Wanders et al., 2013). Trotta et al. (2019) found that the levels of retinal HCA2 receptor protein and apoptosis of retinal cells were increased in in diabetic mice, while the treatment with BHB activated HCA2 and reduced apoptosis, which alleviated retinal damage. Treatment with BHB inhibited retinal ERS by decreasing the levels of the pPERK, pIRE1, and ATF-6 α and suppressed NLRP3 inflammasome activation by reducing the levels of NLRP3 inflammasome, IL-1β, and IL-18 in diabetic mice (Trotta et al., 2019). Moreover, NLRP3 inflammasome is stimulated by ERS and involved in retinal damage in diabetes (Rathinam et al., 2012; Chen et al., 2018). Therefore, it can be inferred that the activation of HCA2 receptor by treatment with BHB improves retinal damage through inhibiting ERS-induced NLRP3 inflammasome, which needs ERS and NLRP3 inflammasome activator to further prove. HCA2 activation may offer a new therapeutic target for treating inflammation in diabetic retinal damage.

## The Role of the Effects of ERS on NLRP3 Inflammasome in Diabetic Nephropathy

Diabetic nephropathy (DN), which is the main cause of end-stage renal disease (Liu L. et al., 2019), is characterized by progressive renal fibrosis, decreased renal function, and irreversible tissue loss (Liu, 2011; Duffield, 2014). Pyroptosis is an inflammatory programmed cell death induced by inflammatory caspase-1 and proinflammatory mediators (Dong et al., 2015; Jorgensen et al., 2016; Man et al., 2017). The activation of NLRP3 inflammasome is closely related to the pyrocytosis, which is involved in diabetes (Wu et al., 2018; Yu et al., 2020). In addition, TXNIP can regulate pyrocytosis through regulating NLRP (Liu X. et al., 2019). Therefore, the inhibition of inflammasome/TXNIP may be a potential treatment for DN. Ke et al. (2020) found that in DN rats, the levels of NLRP3 inflammasome, TXNIP, the pyroptosis-related protein were increased, while silencing TXNIP by lentivirus vector reversed the changes, suggesting that the inhibition of TXNIP/NLRP3 improved renal injury through inhibiting pyroptosis in DN rats. Evidence indicated that ERS regulate pyroptosis through TXNIP/NLRP3 (Chen et al., 2018; Dong et al., 2020). In DN rats, ERS was activated by increasing the levels of CHOP and ATF4 and the phosphorylation of IRE1α. Inhibition of IRE1α could increase miR-200a expression, which downregulated TXNIP levels, indicating that IRE1α mediated the regulatory role of ERS on pyroptosis through TXNIP/NLRP3. The similar results were obtained in HG-induced NRK-52E cell. In sum, the inhibition of IRE1α-mediated ERS can improve DN by downregulating TXNIP/NLRP3 inflammasome-mediated pyroptosis (Ke et al., 2020). In addition to IRE1α, PERK is also an ERS-related protein. Whether the suppression of PERK can downregulate TXNIP/NLRP3 inflammasome-mediated pyroptosis needs to be elucidated. The mechanisms of the relationship between ERS and pyroptosis are complex, which deserves to be further studied.

## Conclusion

**FIGURE 1:**
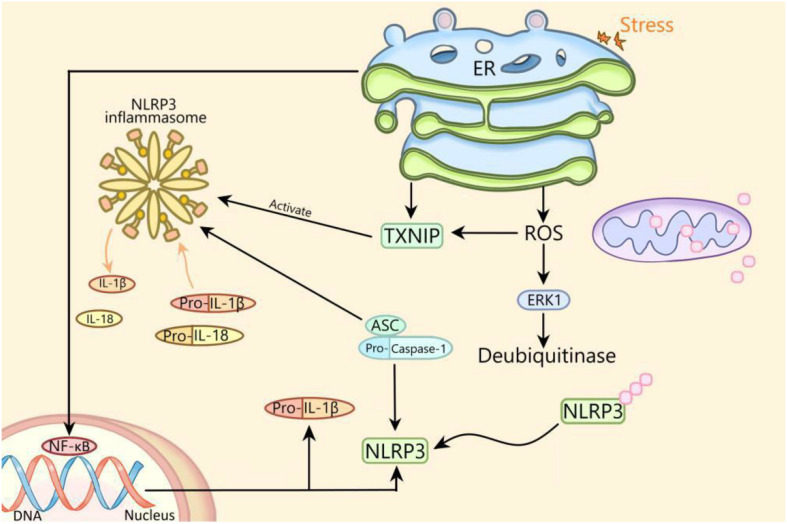
The mechanism of activation of NLRP3 inflammasome by endoplasmic reticulum stress. ER, endoplasmic reticulum; NLRP3, NLRP3 inflammasome; ASC, apoptosis-related spot-like protein containing caspase recruitment domain; ERK1, extracellular signal regulated kinase 1; NF-κB, nuclear factor kappa B; ROS, reactive oxygen species; TXNIP, thioredoxin interacting protein; IL, interleukin.

There is an interaction between ERS and NLRP3 inflammasome. Previous studies have shown that ERS can activate NLRP3 inflammasome through ROS/ERK1 pathway, ROS/TXNIP pathway and NF-κB pathway (Figure 1; Ji et al., 2019). In this review, ERS activates NLRP3 inflammasome through the ROS/TXNIP pathway in diabetes. Whether ERS can activate NLRP3 inflammasome through the ROS/ERK1 pathway or NF-κB pathway and whether NLRP3 inflammasome can regulate ERS in diabetes deserved to be studied. In addition, whether ERS can regulate NLRP3 inflammasome through other ways remains to be further elucidated.

It has been reported that ERS and NLRP3 inflammasome are both the regulative targets of H_2_S (Wang et al., 2020a,b) and involved in diabetes (Ji et al., 2019). Therefore, whether H_2_S can improve diabetes through regulating ERS/NLRP3 inflammasome is a subject worth studying. It is believed that the in-depth study of the relationship between ERS and NLRP3 in diabetes will provide a new strategy for the treatment of diabetes.

## Author Contributions

All authors listed have made a substantial, direct and intellectual contribution to the work, and approved it for publication.

## Conflict of Interest

The authors declare that the research was conducted in the absence of any commercial or financial relationships that could be construed as a potential conflict of interest.
